# The role of histone H3 leucine 126 in fine-tuning the copper reductase activity of nucleosomes

**DOI:** 10.1016/j.jbc.2024.107314

**Published:** 2024-04-22

**Authors:** Nataliya P. Tod, Maria Vogelauer, Chen Cheng, Ansar Karimian, Stefan Schmollinger, Dimitrios Camacho, Siavash K. Kurdistani

**Affiliations:** 1 Department of Biological Chemistry, David Geffen School of Medicine, University of California Los Angeles, Los Angeles, California, USA; 2 Department of Molecular and Cell Biology, University of California, Berkeley, California, USA; 3 Department of Pathology and Laboratory Medicine, David Geffen School of Medicine, University of California Los Angeles, Los Angeles, California, USA; 4 Eli and Edythe Broad Center of Regenerative Medicine and Stem Cell Research, David Geffen School of Medicine, University of California Los Angeles, Los Angeles, California, USA

**Keywords:** chromatin, histone, copper, reductase, yeast

## Abstract

The copper reductase activity of histone H3 suggests undiscovered characteristics within the protein. Here, we investigated the function of leucine 126 (H3L126), which occupies an axial position relative to the copper binding. Typically found as methionine or leucine in copper-binding proteins, the axial ligand influences the reduction potential of the bound ion, modulating its tendency to accept or yield electrons. We found that mutation of H3L126 to methionine (H3L126M) enhanced the enzymatic activity of native yeast nucleosomes *in vitro* and increased intracellular levels of Cu^1+^, leading to improved copper-dependent activities including mitochondrial respiration and growth in oxidative media with low copper. Conversely, H3L126 to histidine (H3L126H) mutation decreased nucleosome’s enzymatic activity and adversely affected copper-dependent activities *in vivo*. Our findings demonstrate that H3L126 fine-tunes the copper reductase activity of nucleosomes and highlights the utility of nucleosome enzymatic activity as a novel paradigm to uncover previously unnoticed features of histones.

As the major DNA-binding proteins in eukaryotes, histones help package DNA into the confines of the nucleus and regulate essentially all DNA-based processes (1). The histones are thought to exert their regulatory influence by altering chromatin structure and undergoing post-translational modifications. These proposed mechanisms have been central to interpreting phenotypes associated with altered histone function. For instance, when assessing the effects of histone mutations discovered through genetic screens, diseases, or genetic variants, researchers typically invoke nucleosome stability, positioning, and changes in modifications to explain potential differences in gene expression between control and experimental conditions (2, 3, 4, 5, 6).

We have recently reported that histone H3 has a catalytic function as an oxidoreductase enzyme, catalyzing the reduction of Cu^2+^ to Cu^1+^ (7). Copper-dependent enzymes throughout the cell, including those in mitochondria, rely on this activity because copper ions must be in their reduced, cuprous (Cu^1+^) state, to be transported to proteins that utilize them as a co-factor (8, 9). Diminishing the copper reductase activity of histone H3 reduced intracellular Cu^1+^ levels without affecting total levels of copper (7). This compromised the function of copper-dependent enzymes such as cytochrome *c* oxidase (CcO - complex IV) of the mitochondrial transport chain (ETC) and superoxide dismutase 1 (Sod1) and resulted in diminished growth, particularly in conditions when demand for copper is high such as in oxidative media. Conversely, the decreased activity of histone H3 increased resistance to copper toxicity (7, 10). This toxicity is mainly due to the high affinity of Cu^1+^ ion for cysteine sulfhydryl groups (11), causing it to compete with iron-sulfur clusters for protein binding sites (12). Although little is known about the regulatory mechanisms of histone H3’s enzymatic activity, maintaining its optimal activity is essential to produce appropriate Cu^1+^ amounts that satisfy cellular needs without surpassing toxicity limits.

We have also recently demonstrated that histone H3 within native yeast nucleosomes retains its enzymatic activity, indicating that eukaryotic DNA is wrapped around an enzyme complex and that chromatin may function as a “metabolic organelle” (13, 14). The enzymatic activity of histone H3 now provides an additional foundation for understanding the potential function of certain histone residues in an entirely new context.

A nucleosome consists of a tetramer of histones H3 and H4, along with two pairs of H2A-H2B dimers, collectively wrapping 146 bp of DNA (15). The histone H3-H4 tetramer is itself composed of two dimers of H3 and H4 histones that interact exclusively through histone H3 residues. The H3-H3′ interface, where the H3-H4 dimers interact, is the probable enzyme active site as it is where Cu^2+^ binds and is likely reduced. The pairs of histidine and cysteine residues at the H3-H3′ interface are arranged in an approximately square planar geometry, similar to Cu^2+^ binding sites in other proteins. Above and below this plane are axial residues that form a cage around the bound Cu^2+^ ion. These axial residues are typically amino acids with hydrophobic side chains such as methionine (M) or leucine (L) and serve to increase the reduction potential of copper—that is, to increase its tendency to accept electrons (16). In general, the degree of axial residue hydrophobicity correlates positively with the reduction potential of the bound copper ion (16). The exact reduction potential of metal ions in proteins must therefore align with the protein's intended role to ensure proper functionality.

Interestingly, occupying the axial position relative to the plane of copper binding in histone H3 is the leucine 126 (H3L126) residue (Fig. 1*A*) (17). The H3L126 residue is highly conserved among eukaryotic and archaeal histones (Fig. 1*B*) yet its potential function has remained a mystery.

**Figure 1:**
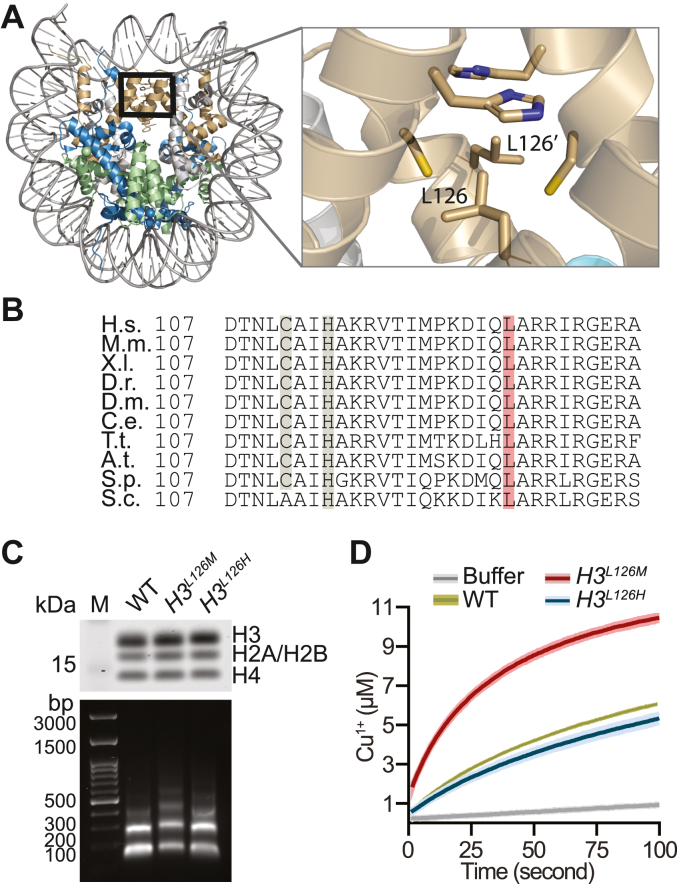
**As the axial ligand for copper binding, H3L126 modulates nucleosome copper reductase activity.***A*, *left*: *X. laevis* nucleosome core particle structure [Protein Data Bank (PDB) 1KX5] (17). The box delineates the H3-H3′ interface. *Right*: H3L126 residues from opposing histone H3 proteins relative to the interface residues H3H113 and H3C110 are shown. *B*, alignment of the C-terminal region of histone H3 from *Homo sapiens* (H.s.), *Mus musculus* (M.m.) *Xenopus laevis* (X.l.), *Danio rerio* (D.r.), *Drosophila melanogaster* (D.m.), *Caenorhabditis elegans* (C.e.), *Tetrahymena thermophila* (T. t.), *Arabidopsis thaliana* (A.t.), *Schizosaccharomyces pombe* (S. p.), and *S. cerevisiae* (S.c.). Note that the numbering excludes the initial methionine residue. *C*, representative examples of protein (*upper panel*) and DNA (*lower panel*) from nucleosomal fractions isolated from the indicated strains. *D*, progress curves of Cu^2+^ reduction by nucleosomes isolated form WT, *H3*^*L126H*^ or *H3*^*L126M*^ strains or buffer. *Lines* and *shading* represent the mean ± standard deviation (SD) of three assays.

Here we present evidence that H3L126 plays a critical role in optimizing the enzymatic activity of nucleosomes. We generated yeast strains carrying the H3L126M and H3L126H mutations. We chose methionine because it frequently serves as an axial ligand in other copper proteins, expecting it to either have no effect or potentially serve as a gain-of-function mutation. The H3L126H mutant would be expected to decrease the hydrophobicity of the Cu^2+^ binding pocket in histone H3, thereby reducing its enzymatic activity. Indeed, *in vitro* experiments confirmed that the H3L126M mutation enhanced the copper reductase activity of native nucleosomes. In cells, H3L126M mutations increased intracellular levels of Cu^1+^ without affecting the total copper content, and improved oxygen consumption, which is mainly due to copper-dependent CcO (Complex IV) activity, as well as growth in oxidative media with limited copper. The H3L126H mutation exerted the opposite effects, diminishing the nucleosome copper reductase activity and compromising respiration and growth in oxidative media, especially under low copper conditions. Analysis of global gene expression revealed minimal changes in response to fluctuations in H3L126 mutations, arguing against major structural effects on chromatin. Our findings identify the H3L126 residue as the potential axial copper ligand in histone H3 and unveil its importance in modulating the copper reductase activity of the nucleosome. Our results further demonstrate how the copper reductase activity of histone H3 provides a novel paradigm to understand nucleosome structure and function, including potentially the contributions of histone mutations to human disease.

## Results

### Mutation of histone H3L126 modulates copper reductase activity of nucleosomes

To determine if H3L126 affects nucleosome activity, we generated yeast strains carrying the H3L126M or H3L126H mutation in both copies of the histone H3 genes without any other genetic perturbation. Modeling these mutations in the published structure of the yeast nucleosome (PDB file 1ID3) (18) revealed steric clashes comparable to those observed for other residues within the structure. The steric clashes for all residues range from 0.4 to 1.12 Å, whereas for M126 they range from 0.46 to 0.54 Å and for H126 from 0.43 to 0.57 Å (Fig. S1). It was also previously reported that it is sterically possible for the H3L126 residue to adopt different conformations, suggesting a degree of flexibility in accommodating steric bulk (15).

Next, we obtained native nucleosomes from wildtype (WT), *H3*^*L126M*^, and *H3*^*L126H*^ strains by micrococcal nuclease digestion of chromatin in isolated nuclei and ion-exchange chromatographic purification (Fig. S2). DNA extraction of the collected aliquots from each strain showed comparable DNA fragments of mostly mono-, di- and tri-nucleosomes, as expected from nucleosomal particle purification (Fig. 1*C*). To test if the isolated nucleosomes reduce Cu^2+^ to Cu^1+^ ions *in vitro*, we performed a copper reduction assay. Assays contained native nucleosomes or control buffer and a reduced form of tetramethyl-1,4-phenylenediamine (TMPD) as a one-electron donor, which upon oxidation turns a characteristic blue-violet color, providing a measure of copper reduction by the nucleosomes. Reactions were initiated by addition of Cu^2+^ in the form of Cu^2+^-ADA (N-(2-Acetamido)iminodiacetic acid) complex. ADA is a zwitterionic organic chemical buffering agent that can form complexes with most common metals (19). Spontaneous Cu^1+^ production occurred at a slow rate, but the rate of Cu^1+^ production substantially increased in the presence of wild-type (WT) nucleosomes (Fig. 1*D*). Production of Cu^1+^ eventually plateaued due to near-full consumption of the electron donor. Nucleosomes isolated from the *H3*^*L126M*^ strain reproducibly demonstrated substantially higher enzymatic activity compared to WT, whereas those from the *H3*^*L126H*^ strain showed modestly diminished activity (Fig. 1*D*). We obtained similar results using Cu^2+^-Tricine as the substrate for the nucleosomes (Fig. S3). These results indicate that the H3L126M and H3L126H mutations respectively increase and decrease the copper reductase activity of native yeast nucleosomes *in vitro*.

It is important to note that our copper reductase assay may not precisely recapitulate the conditions inside the cell. One notable factor is our lack of knowledge regarding the specific chaperones responsible for delivering Cu^2+^ to nucleosomes or transporting Cu^1+^ away from them. The identities of these chaperones could potentially influence the magnitude of the observed differences between WT and mutant nucleosomes. We find it noteworthy that despite the empirical condition of our assay, we still observe a discernible difference between WT and mutant nucleosomes. Nevertheless, we urge caution when drawing direct proportional comparisons between the *in vitro* and *in vivo* effects, given the likely differences in the enzymatic milieu.

### Mutation of histone H3L126 regulates intracellular Cu^1+^ levels

**Figure 2:**
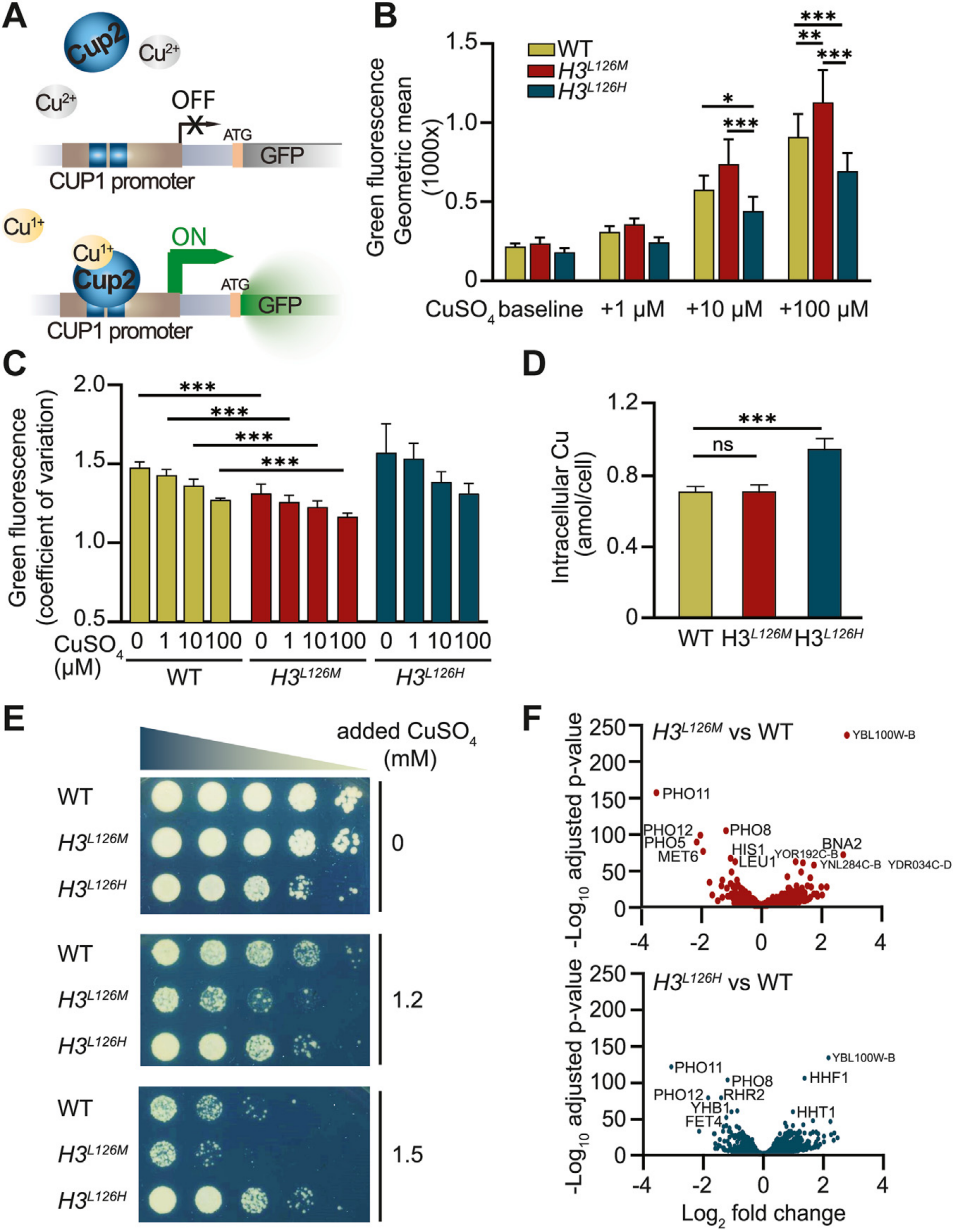
**Mutations of H3L126 modulate intracellular copper oxidation state.***A*, graphic representation of the reporter assay used to determine intracellular Cu^1+^. *B*, geometric mean green fluorescence signal of WT, *H3*^*L126M*^, or *H3*^*L126H*^ cells containing the p^(CUP1)^-GFP plasmid grown in oxidative media without (0) or with 1, 10 or 100 μM additional CuSO_4_ from six experiments. The baseline copper concentration is ∼0.16 μM. *C*, coefficient of variation (CV) in green fluorescence signal of WT, *H3*^*L126M*^, or *H3*^*L126H*^ cells without (*baseline*) or with 1, 10 or 100 μM additional CuSO_4_ from six experiments. The baseline copper concentration is ∼0.25 μM. *D*, intracellular copper content of exponentially growing strains as determined by ICP-MS/MS. Bars are means ± SD from four technical repeats of five independent experiments. *E*, spot test assays in SC media without or with 1.2 or 1.5 mM additional CuSO4. Copper concentration in at baseline is ∼0.25 μM. *F*, volcano scatter plots of average log_2_ fold changes in expression of genes from *H3*^*L126M*^*versus* WT (upper panel) or *H3*^*L126H*^*versus* WT (lower panel) strains. Genes with an expression change significance greater than 50 -log_10_ adjusted *p*-value are indicated. Statistically significant differences as determined by two tailed *t* test are indicated by *asterisks*: ∗*p* < 0.05, ∗∗*p* < 0.01, ∗∗∗*p* < 0.001. ns, not significant.

To provide *in vivo* evidence for the potential effects of the H3L126M and H3L126H mutations on the enzymatic activity of nucleosomes, we used a plasmid to report on the activity of the Cup2 transcription factor, which is activated directly by Cu^1+^ and not Cu^2+^ ions (Fig. 2*A*). The reporter plasmid contains the green fluorescent protein (GFP) gene downstream of the CUP1 promoter, a main target gene of Cup2 (20, 21). WT cells containing this plasmid show an increasing amount of Cu^1+^ intracellularly with increasing amounts of Cu^2+^ provided in the growth medium (Figs. 2*B* and S4*A*). However, the GFP expression in the *H3*^*L126M*^ strain increased significantly more than in WT cells with increasing amounts of copper in the medium, indicating that this mutation improved the conversion of Cu^2+^ to Cu^1+^ intracellularly. Conversely, the *H3*^*L126H*^ strain produced less Cu^1+^ than the WT with additional exogenous copper (Figs. 2*B* and S4*A*).

We also observed a decrease in the coefficient of variation (CV) of the GFP signal, a measure of dispersion around the mean, in WT cells when the growth medium was supplemented with increasing amounts of copper (Fig. 2*C*). This observation suggests that the addition of exogenous copper leads to enhanced uniformity in the intracellular levels of Cu^1+^ within the cell population. Importantly, the CV of the GFP signal from the *H3*^*L126M*^ strain was significantly lower than that of WT in medium with a baseline amount of copper (∼0.25 μM), down to a level comparable to WT cells exposed to 100 μM copper (Fig. 2*C*). The CV of the GFP signal from the *H3*^*L126M*^ strain decreased even further by additional exogenous copper. The CV in the GFP signal from the *H3*^*L126H*^ strain was similar to WT and decreased in response to 100 μM exogenous copper to comparable levels as the *H3*^*L126M*^ strain in medium with baseline copper levels (Fig. 2*C*). These findings suggest that in a yeast population, an increase in exogenous copper levels results in an elevated Cu^1+^ production within the cells, thereby reducing the variability in intracellular Cu^1+^ levels. The observation that the H3L126M mutation decreases the variability in intracellular Cu^1+^ levels across the population at any given copper concentration compared to WT is consistent with the H3L126M mutation acting to enhance the nucleosome copper reductase activity.

We utilized Inductively Coupled Plasma Mass Spectrometry (ICP-MS/MS) to measure the intracellular copper content in the WT and both mutant strains. This was to ensure that the fluctuations in Cu^1+^ levels were not merely reflective of overall cellular copper content alterations. The *H3*^*L126M*^ strain demonstrated no significant change in its copper content relative to the WT (Fig. 2*D*), indicating that the rise in Cu^1+^ is solely from improved intracellular conversion. Conversely, the *H3*^*L126H*^ strain displayed a statistically significant increase in total intracellular copper content (Fig. 2*D*), accentuating its diminished ability to convert Cu^2+^ into Cu^1+^. These findings suggest that the elevated Cu^1+^ levels in *H3*^*L126M*^ result from alterations in the Cu^1+^/Cu^2+^ ratio, rather than overall copper content changes. For *H3*^*L126H*^, the increase in total intracellular copper might represent an adaptive response to compensate for the reduced Cu^1+^ production.

Next, we hypothesized that variations in intracellular Cu^1+^ levels resulting from H3L126 mutations could influence resistance to Cu^1+^ toxicity. On media containing toxic levels of copper, *H3*^*L126M*^ exhibited poorer growth than WT, whereas *H3*^*L126H*^ showed better growth (Fig. 2*E*). These findings are consistent with the respective shifts in intracellular Cu^1+^ levels observed in the H3L126 mutant strains.

The observed differences in Cu^1+^/Cu^2+^ ratio were not due to altered expression of genes involved in metal homeostasis, as minimal variations were found in the global gene expression patterns of WT *versus H3*^*L126M*^ or *H3*^*L126H*^ cells, as assessed by mRNA-seq (Figs. 2*F* and S4*B*). Neither mutant strains exhibited substantial changes in global expression as shown in the volcano plots (Fig. 2*F*). Although a few genes were up- or downregulated in the mutant strains as indicated in Figure 2*F*, there were no statistically significant gene ontology terms in the molecular function category for genes showing two-fold upregulation in *H3*^*L126M*^ or *H3*^*L126H*^ *versus* WT. A minor enrichment in the GO terms “acid phosphatase activity” and “phosphoric ester hydrolase activity” was observed for two-fold downregulated genes in *H3*^*L126M*^ and *H3*^*L126H*^, respectively (Figs. 2*F* and S4*B*). However, since these genes are downregulated similarly in both mutants, it is unlikely that their altered expression is the cause of the observed copper-related phenotypes.

Altogether, these findings suggest that consistent with the *in vitro* enzymatic assays, the H3L126M and H3L126H mutations, respectively, increase and decrease intracellular Cu^1+^ levels. Because the reporter output is transcriptional, these data furthermore indicate that the changes in Cu^1+^ levels occur within the nuclei of cells.

### Mutation of histone H3L126 affects mitochondrial oxygen consumption and oxidative growth

**Figure 3:**
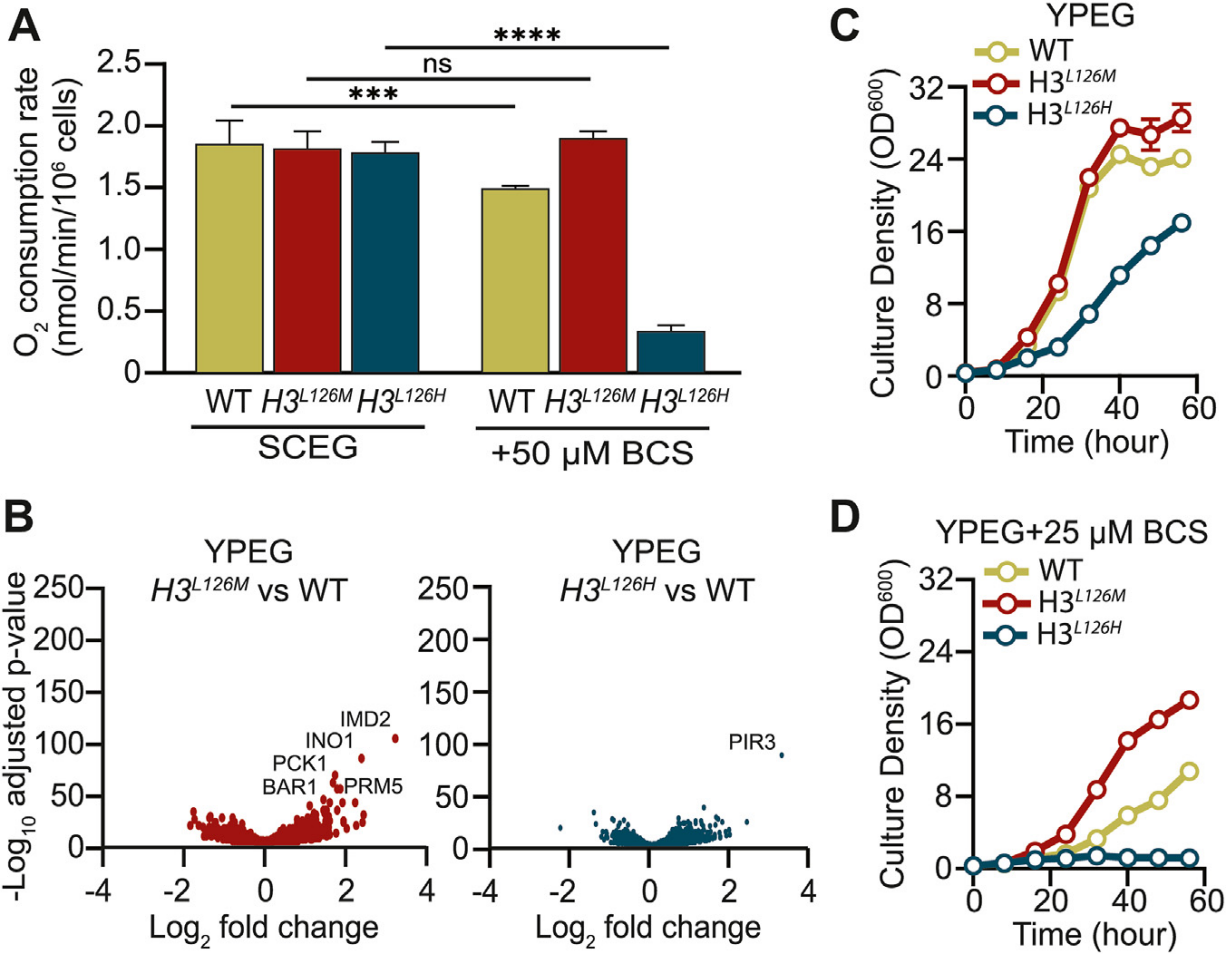
**Mutations of H3L126 modulate mitochondrial respiration and oxidative growth.***A*, oxygen consumption assays of exponentially growing WT, *H3*^*L126M*^, or *H3*^*L126H*^ cells in oxidative media in the absence or presence of the BCS Cu^1+^ chelator. Bars are means ± SD from 4 to 6 experiments. Statistically significant differences as determined by two tailed *t* test are indicated by asterisks: ∗∗∗*p* < 0.001, ∗∗∗∗*p* < 0.0001. ns, not significant. *B*, volcano scatter plots of average log_2_ fold changes in expression of genes from *H3*^*L126M*^*versus* WT (*left*) or *H3*^*L126H*^*versus* WT (*right*) strains grown in oxidative media. Genes with an expression change significance above 50 -Log_10_ adjusted *p*-value are indicated. *C* and *D*, growth curves of WT, *H3*^*L126M*^, or *H3*^*L126H*^ cells in oxidative media without or with 25 μM the BCS Cu^1+^ chelator. *Lines* show means at each time point ± SD from four experiments (note that the SD bars on certain data points may be too small and obscured).

Mitochondrial oxygen consumption depends on the activity of the copper-dependent cytochrome *c* oxidase, which is activated in oxidative media (22). Therefore, we measured mitochondrial respiration in oxidative media to determine the functional impacts of H3L126 mutations. In a medium where the main sources of carbon are ethanol and glycerol, and cellular metabolism relies on mitochondrial oxidative phosphorylation, with a baseline copper level of ∼0.25 μM, no difference was observed in oxygen consumption rates between WT, *H3*^*L126M*^, and *H3*^*L126H*^ strains. However, in the presence of the copper chelator bathocuproinedisulfonic acid (BCS), which limits copper availability to cells, there were significant decreases in oxygen consumption rates of WT cells, and more dramatically, of *H3*^*L126H*^ cells (Fig. 3*A*). In contrast, *H3*^*L126M*^ cells exhibited consistent levels of oxygen consumption between baseline and the low copper conditions (Fig. 3*A*). To determine if these sensitivities were due to changes in the expression of genes involved in mitochondrial ETC, we performed global gene expression analyses using mRNA-seq of cells growing in oxidative media. As shown in the volcano scatter plots of Figure 3*B*, there were little significant differences in global gene expression patterns of WT *versus H3*^*L126M*^ or *H3*^*L126H*^ cells (Figs. 3*B* and S5*A*), or in the expression of genes encoding the components of the mitochondrial electron transport chain (Fig. S5*B*).

We also analyzed the expression of Cox2, subunit II of cytochrome *c* oxidase (Complex IV), by Western blotting using cells grown in the same conditions as those used for measuring O_2_ consumption (Fig. S6*A*). Our analysis revealed modest but statistically significant differences in Cox2 levels in cells grown in oxidative media, with L126M and L126H mutations resulting in increased and decreased levels, respectively, compared to WT (Fig. S6*A*). When cells were grown in oxidative media with limited copper, we again observed a modest but statistically significant decrease in Cox2 protein levels in WT and L126M strains, but not in the L126H strain, which initially exhibited the lowest levels. Notably, under this condition, all three strains exhibited comparable levels of Cox2 protein, indicating that the differences in oxygen consumption cannot be attributed to changes in Cox2 protein levels. Furthermore, the total intracellular levels of copper were comparable between all strains (Fig. S6*B*). These data suggest that, compared to WT cells, *H3*^*L126M*^ cells exhibit greater resistance to functional impairments under conditions of low copper availability likely due to an increased ratio of Cu^1+^/Cu^2+^ and enhanced intracellular copper utilization. In contrast, the *H3*^*L126H*^ cells, characterized by a decreased ratio of intracellular Cu^1+^/Cu^2+^, display the opposite phenotype.

To relate the defects in mitochondrial respiration to cellular phenotypes, we assessed the growth of the three strains in oxidative media. The *H3*^*L126M*^ cells grew similarly to WT cells while the *H3*^*L126H*^ cells grew much slower in the medium with baseline levels of copper (Fig. 3*C*). In the presence of BCS to limit copper in otherwise oxidative media, the growths of all strains were hindered but much less so for the *H3*^*L126M*^ cells compared to WT and *H3*^*L126H*^ cells, which were most severely affected (Fig. 3*D*). Altogether, these findings suggest that the *H3*^*L126M*^ cells, by virtue of their enhanced capacity to produce Cu^1+^, are able to effectively sustain mitochondrial respiration and support oxidative growth in low copper environments when compared to the WT cells. Conversely, the decreased ability of *H3*^*L126H*^ cells to produce Cu^1+^ renders them susceptible to copper scarcity, leading to a significantly impaired ability to sustain optimal mitochondrial function compared to the WT cells.

### The H3L126M mutation supports copper utilization by mitochondrial and cellular processes and pathways

**Figure 4:**
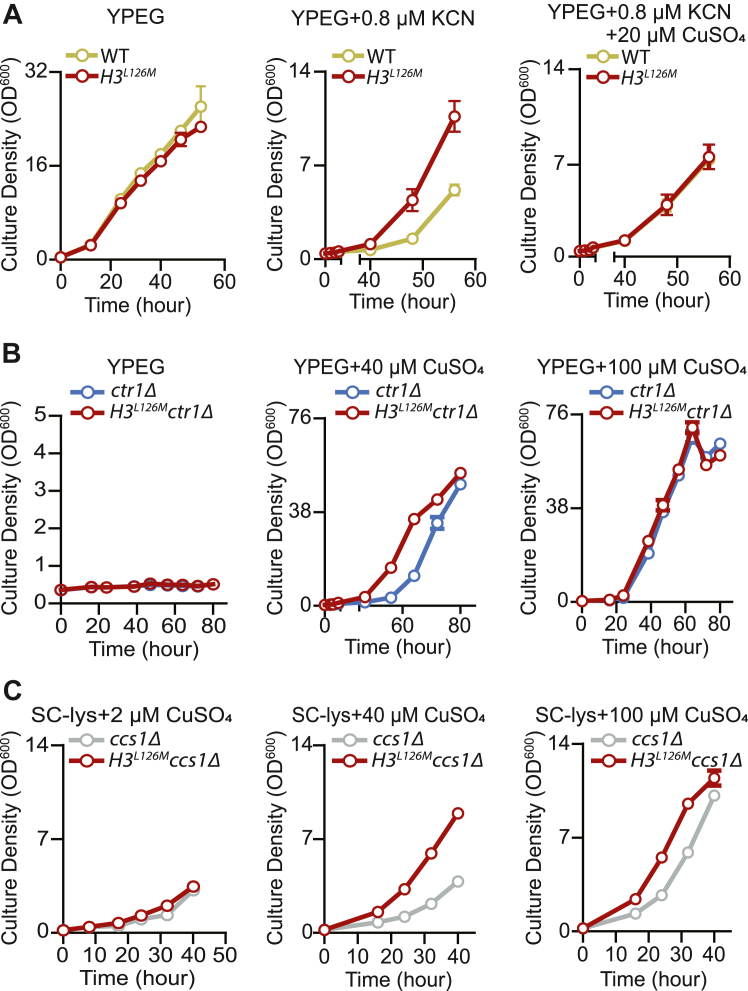
**The H3L126M mutation enhances copper utilization in *S. cerevisiae*.***A*, growth curves in oxidative media of the WT and *H3*^*L126M*^ strains without and with potassium cyanide (KCN) or with KCN and additional CuSO_4_ as indicated. *Lines* show means at each time point ± SD from three or four experiments. *B*, growth curves in oxidative media of the *ctr1Δ* and *ctr1ΔH3*^*L126M*^ strains without and with additional CuSO_4_ as indicated. Lines show means at each time point ± SD from four experiments. *C*, growth curves in SC lacking lysine of the *ccs1Δ* and *ccs1ΔH3*^*L126M*^ strains without and with additional CuSO_4_ as indicated. Note that yeast cells lacking Ccs1 or Sod1 function are auxotrophic for lysine. *Lines* show means at each time point ± SD from four experiments (note that the SD bars on certain data points may be too small and obscured).

We next sought to provide independent, complementary evidence that the H3L126M mutation supports copper utilization by the mitochondria. First, the *H3*^*L126M*^ cells were more resistant than WT cells to a sublethal amount of potassium cyanide (KCN), an inhibitor of CcO, while this difference was abrogated by supplementation of additional exogenous copper (Fig. 4*A*). Second, deletion of *CTR1* (*ctr1Δ*), the main copper importer in yeast, impedes growth in oxidative media, which is due to insufficient intracellular copper as increasing supplementation with exogenous copper rescues the growth defect (Fig. 4*B*). Consistent with previous results, the H3L126M mutation enhanced the growth of *ctr1Δ* cells further at lower levels of exogenous copper, compared to cells with WT histone H3 (Fig. 4*B*). Altogether, the increased intracellular Cu^1+^ levels, superior oxygen consumption, improved growth in oxidative media, and resistance to KCN when intracellular copper levels are low, confirm that the H3L126M mutation serves as a gain-of-function mutation that improves copper utilization by the mitochondria.

Finally, we determined if the H3L126M mutation improves copper utilization by proteins other than mitochondrial Complex IV. An important copper-dependent protein is Sod1, which relies on Copper Chaperone for SOD1 (Ccs1) for the acquisition of its copper co-factor (23). In yeast, loss of function of Sod1 or Ccs1 causes lysine auxotrophy presumably due to damage to iron-sulfur cluster containing proteins involved in lysine biosynthesis (24, 25). Correspondingly, both *ccs1Δ* and *H3*^*L126M*^ *ccs1Δ* cells showed minimal growth in a lysine-deficient medium (Fig. 4*C*). However, supplementation of growth media with increasing amounts of copper led to improved growth of *H3*^*L126M*^ *ccs1Δ* cells with *ccs1Δ* cells lagging behind. These data indicate that the H3L126M mutation substantially facilitates copper-dependent recovery of Sod1 function to restore the growth of *ccs1Δ* cells in lysine-deficient conditions (Fig. 4*C*).

## Discussion

Histones were initially understood to solely be structural components of the eukaryotic genome with the primary function of packaging large amounts of DNA within the nucleus. Pioneering experiments in yeast during the 1980s and 90s revealed that histones also function in regulating gene expression, laying the foundation for the model that a system of “writers, readers and erasers” establish and implement the effects of post-translational modifications (26). The ability of histones to modify chromatin structure and serve as platforms for recruiting protein complexes has been the predominant framework for interpreting the phenotypes associated with changes in histone function over the past 3 decades. This epigenetic framework also encompasses mutations in histones, including histone H3, which are increasingly being recognized for their involvement in human diseases such as developmental disorders and cancer (2, 3, 4, 5, 6). It is generally thought that the common route for these mutations in histones leading to abnormal gene expression is by affecting epigenetic regulatory patterns at specific genes in specific cells, ultimately resulting in disease (2, 3, 4, 5, 6).

However, the epigenetic framework may not fully explain the evolution of a histone-based DNA binding system in archaea, the contribution of histones to eukaryogenesis or certain features of modern eukaryotic nucleosomes. The discovery of the role of histone H3 in copper metabolism now provides an additional metabolic context to understand chromatin structure and function. For example, studying the function of H3L126 within the epigenetic framework would involve linking it to potential effects on chromatin structure or histone modifications that impact gene expression. This line of reasoning would also apply to understanding the mutations of H3L126 to methionine, isoleucine, valine, and phenylalanine reported in The Cancer Genome Atlas (TCGA) or other histone mutations (27).

We propose that the enzymatic activity of histone H3 as a copper reductase enzyme suggests that certain aspects of the protein and the larger histone-DNA complex were previously overlooked. The enzymatic activity of histone H3, therefore, provides an additional framework to identify and understand these features and their alterations in human disease.

In this report, we present evidence for one such feature that became apparent in light of histone H3's copper reductase activity. A seemingly unremarkable residue, H3L126 occupies the axial position for copper binding by histone H3. Studies of the axial ligands in other copper proteins have revealed that the nature of the residue, and specifically its hydrophobicity, affects the reduction potential of copper (16). The redox potential of metal ions in proteins is crucial and must be compatible with the protein's function to ensure proper electron transfer in the intended direction and to the correct destination. In the case of histone H3 and the nucleosome, electrons must transfer from an electron donor co-factor to the protein and travel to the Cu^2+^ ions at the active site, rather than other regions of the protein. Therefore, the reduction potential of the bound Cu^2+^ must be more positive than the electron-donating co-factor and other regions within the protein-DNA complex. Additionally, the reduction potential of the binding pocket could affect the histone H3's affinity to capture and bind Cu^2+^ from other chaperones. Since even subtle variations in the coordination environment can influence this, the reduction potential of protein-bound metals must be finely tuned to achieve the correct functional output (28).

Our findings suggest that H3L126 plays an important role in regulating the enzymatic activity of the nucleosome. Whether H3L126 serves a comparable function as a *bona fide* axial ligand in histone H3 will require direct measurement of the reduction potential of Cu^2+^ ions bound at the active site. However, based on its location and the data presented here, H3L126 can be nominated as a potential axial ligand that establishes the desired reduction potential of histone H3-bound Cu^2+^ ions, akin to other copper-binding proteins. The precise factors and biological reasons behind the choice of leucine as the potential axial ligand in histone H3 and the preferred reduction potential remain to be determined. Nevertheless, our data provide a rationale for the reported cancer-associated mutations in H3L126, all of which consist of relatively hydrophobic residues. These mutations could subtly alter the reduction potential of Cu^2+^ ions to meet the cancer cell need for Cu^1+^ ions, adapt to the broader redox landscape of the cell, or respond to the multitude of other factors that may necessitate re-adjusting the copper reductase activity of nucleosomes. Whatever the case may be, our findings demonstrate how the enzymatic activity of the nucleosome can reveal new features and potentially explain certain alterations of histones in human illness.

## Experimental procedures

### Strain generation and growth conditions

All *S.* cerevisiae strains used in this study are based on OCY1131, which was generated from BY4741 (S288C background, MATa) (7). Strains are listed in Table S1. The CRISPR-Cas9 system, optimized for *Schizosaccharomyces cerevisiae,* was used to generate the H3L126M and H3L126H mutations in both chromosomal loci (HHT1 and HHT2) and to delete *CTR1* (29). The guide RNA sequence to target *HHT1* L126 was *AAGTTGGCTAGAAGATTAAG*GTTTTAGAGCTAGAAATAGCAAGT, to target *HHT2* L126 was *AAATTGGCCAGAAGACTAAG*GTTTTAGAGCTAGAAATAGCAAGT and to target *CTR1* was *TATGGGTAGCAGCATGAATA*GTTTTAGAGCTAGAAATAGCAAGT (gene-specific sequences are in italic and underlined). *CCS1* was deleted by standard yeast gene replacement and targeted insertion methodology using selectable marker integration (30, 31). All strains are routinely maintained on YPD (1% yeast extract, 2% peptone, and 2% D-glucose) plates. In addition, strains with *CTR1* deleted were maintained on YPD plates supplemented with 20 μM CuSO_4_ before experiments to prevent accumulation of defects in *ctr1Δ*, therefore improving the reproducibility of experiments. Fermentative media were SC (SC medium with 2% glucose, all amino acids, and uracil and adenine), SC lacking lysine (SC-lys), or SC lacking uracil (SC-ura). Non-fermentative media were either YPEG (1% Yeast Extract, 2% Peptone, 2% ethanol, 2% glycerol) or SCEG (SC except with 2% ethanol and 2% glycerol as carbon source). All strains were grown at 30 °C in all experiments. To prevent contamination with trace metals all glassware was treated with 3.7% hydrochloric acid for at least 12 h followed by at least 12 h of 10% nitric acid.

### Purification of recombinant lyticase

A plasmid for the expression of recombinant lyticase was kindly provided by Craig Peterson (pCP330). Expression and purification of recombinant lyticase was performed as described in (14).

### Nucleosome purification

All necessary glassware was treated with 3.7% hydrochloric acid for 24 h followed by 16 h of 10% nitric acid to remove trace metal contamination. All solutions, buffers, and washes were prepared using Milli-Q (Millipore Sigma) ultra-pure water. Solutions were prepared using BioUltra grade (Sigma) reagents when available. The remaining trace metals were removed with Chelex 100 (Sigma). *S. cerevisiae* cells were grown to OD_600_∼1.5, washed once with water, and resuspended in 5 ml/g cells preincubation solution (60 mM β–mercaptoethanol, 100 mM Tris-HCl pH 8) and incubated for 15 min at room temperature under constant agitation. Cells were resuspended in 5 ml/g cells lyticase buffer (10 mM β–mercaptoethanol, 0.7 M Sorbitol, 0.75% Yeast Extract, 1.5% Bacto Peptone, 10 mM Tris-HCl pH7.5) and the cell wall was digested recombinant lyticase at 30 °C for 30 min followed by one wash with 1 M Sorbitol. Spheroplasts were resuspended in 5 ml/g cells Ficoll Solution (18% Ficoll, 20 mM KH_2_PO_4_ pH 7.5, 1 mM MgCl2, 0.5 mM EDTA, 1x Protease inhibitors cocktail (Roche)) for cell lysis. Crude nuclei were recovered by centrifugation at 30,000 rcf for 30 min and resuspended in 4 ml/g of cells in a Digestion Buffer (1 M Sorbitol, 10 mM Tris-HCl pH7.5, 50 mM NaCl, 1 mM CaCl_2_, 5 mM MgCl_2_, 1 mM β–mercaptoethanol and 1x Protease inhibitors cocktail). Crude nuclei were reacted with 8 U/g cells MNase (Sigma) at 37 °C for 30 min. The reaction was quenched by the addition of one-fourth volume of 5x Quenching Buffer (40 mM Tris-HCl pH 7.5, 2.45 M NaCl, 62.5 mM EDTA) and incubated on ice for 15 min to release chromatin fragments into solution. Chromatin was then cleared by centrifugation at 30,000 rcf for 30 min. The supernatant containing chromatin fragments was then treated with 15 μg/g cells DNase-free RNase (Roche) at 37 °C for 90 min and again clarified by centrifugation at 30,000 rcf for 30 min. The cleared chromatin extract was then loaded recursively onto a Q HP HiTrap (Cytiva) at 4 °C for 16 h. The loaded Q HP HiTrap was then washed with 20 column volumes of Buffer A (5 mM Tris-HCl pH 7.5, 300 mM NaCl) and nucleosomes were eluted using a 12-column volume salt gradient to reach 100% of Buffer B (5 mM Tris-HCl pH 7.5, 750 mM NaCl). 1 ml fractions containing eluted proteins were analyzed by polyacrylamide gel electrophoresis (PAGE). Gels were stained with SimplyBlue Safe Stain (Invitrogen) and scanned using Odyssey DLx imager (Licor). Nucleosomal fractions were pooled and concentrated using an Amicon Ultra 15 with a molecular weight cut off of 50K (Millipore). Salt concentration was then reduced twice by stepwise addition of 20 volumes of 5 mM Tris-HCl pH7.5, 50 mM NaCl followed by further concentration. The resulting final flow-through was used as “buffer” control in subsequent experiments.

### Copper reduction assay

0.5 μM of nucleosomes or an equivalent volume of control buffer were incubated with 25 or 50 μM TMPD, as indicated. Reactions were started by adding substrate in the form of a CuSO_4_-ADA pH7.5 (Sigma) 1:1 or CuSO_4_-Tricine pH7 (Sigma) 1:8 mix, as indicated, at a final reaction concentration of 0.2 mM. The appearance of Cu^1+^ was then measured at 611 nm every 0.5 s using a Hewlett-Packard HP8453 diode-array UV/Visible spectrophotometer and absorbance was converted to μM Cu^1+^ using an extinction coefficient of 12,200 M^−1^ cm^−1^.

### Cup2 reporter assay and flow cytometry analysis

The Cup2 reporter plasmid was introduced into yeast strains by a standard transformation procedure as described previously (7). Briefly, single colonies of cells bearing the Cup2 reporter plasmid were grown in liquid SC-ura media to exponential phase and OD_600_ ∼0.5 to 1. The green fluorescence signal was determined by flow cytometry using a BD LSRFortessaX-20 instrument. Background signal was determined using cells without GFP expression and gated out. Replicate experiments were from different clones from the same transformation. A minimum of 30,000 events were analyzed for each clone and the average geometric mean and covariance of six independent transformants are reported.

### Inductively-coupled plasma mass spectrometry (ICP-MS)

Cells from logarithmically growing cells were collected and washed twice in 1 mM EDTA for removal of extracellular metals and once in water. Cell pellets were frozen and stored at −20 ^o^C until further processed for ICP-MS/MS. Cell pellets were overlaid with 70% nitric acid and digested at room temperature for 24 h, followed by incubation at 65 °C for at least 2 h, before being diluted to a final nitric acid concentration of 2% (v/v). Inductively coupled mass spectrometry was performed on the Agilent 8900 ICP-MS/MS. ^63^Cu were used to determine total cellular copper levels using He as a cell gas, ^89^Y as an internal standard (Inorganic Ventures MSY-100PPM), and calibrated with an environmental calibration standard (Agilent 5183-4688). All measurements were within the calibrated linear ranges. The average of four technical replicate measurements was used from five independent biological samples where the technical variation never exceeded 5% for any individual sample. ICP-MS/MS data were then analyzed using the Agilent ICP-MS MassHunter software (v4.4).

### Oxygen consumption assay

Cells were grown in SC overnight, washed twice with 5 mM EDTA to remove excess metals possibly adhered to the cell wall, rinsed once in water, and diluted in SCEG, with or without 50 μM BCS, and grown at 30 °C for four to 12 h, respectively (note that BCS slows cell growth). Oxygen consumption rates of whole cells were measured using the Fiber Optic Oxygen Monitor, Model 110 (Instech Laboratories Inc). Before assessing oxygen consumption, cells were diluted to OD_600_ of three in oxidative media. For measurements, an OD_600_ of one was used for cells without BCS, and an OD_600_ of 1.5 for those with BCS—the elevated OD with BCS ensured linearity in the data. Consumption was recorded for 5 min and the rate was determined from a one-minute linear consumption period.

### Western blotting for Cox2p

Cells were grown as described for “Oxygen consumption assay”. 2 × 10^8^ cells were resuspended in 0.8 ml of 200 mM NaOH and incubated at room temperature for 5 min. Cells were collected by centrifugation and the pellet was resuspended in 0.15 ml loading buffer and boiled for 5 min 10 μl of each sample was used for polyacrylamide gel electrophoresis (PAGE), followed by western blotting onto Immobilon PVDF membrane (0.2 μm) (Millipore). The resulting membranes were probed with antibodies specific for either yeast Cox2p (anti-MTCO2; Abcam ab110271) at a dilution of 1:500 or for yeast Act1p (anti-β-Actin; Abcam ab170325) at a dilution of 1:5000. Secondary antibodies were IRDye800CW and IRDye680RD (Licor). Proteins were visualized using Odyssey DLx imager (Licor) and signal intensity was analyzed using the provided software (Empiria Studio–Licor).

### RNA-seq and differential gene expression analysis

Cells grown in fermentative (YPD) or oxidative media (YPEG) were collected by centrifugation and frozen at −20 °C until processed for RNA extraction and RNA-seq. RNA was extracted using previously published methods (32). RNA extracted for subsequent RNA-seq analysis is from four replicate experiments. Contaminating DNA was digested using Turbo DNase (Thermo Fisher Scientific). Sequencing libraries were prepared and sequenced by the UCLA Technology Center for Genomics & Bioinformatics (TCGB). High-throughput sequencing was performed on Illumina’s NovaSeq system. The total read count per library ranged from ∼8 to 15 million. Demultiplexed reads, in FASTQ file format, were aligned in a strand-specific manner to the R64-1-1 S288C reference genome assembly (sacCer3) using HISAT2 (33). Assigning and counting reads for 6692 annotated open reading frames were performed using featureCounts (34). Determination of adjusted *p* values for differential gene expression comparisons was done using DESeq2 (35). For iPAGE analysis in Fig. S5*A*, normalized reads for each strain were used as input, and a *p*-value cutoff of 0.005 was used for analysis (https://tavazoielab.c2b2.columbia.edu/iPAGE/) (36).

## Data availability

Gene expression datasets are available on the NCBI GEO database (GSE243833).

## Supporting information

This article contains supporting information (37).

## Conflict of interest

The authors declare that they have no conflicts of interest with the contents of this article.
