# Supplementary material for: The role of histone H3 leucine 126 in fine-tuning the copper reductase activity of nucleosomes
Source: J Biol Chem. 2024 Apr 22;300(6):107314. doi: 10.1016/j.jbc.2024.107314 (PMC11134540; doi:10.1016/j.jbc.2024.107314)
Supplement: Supporting Information [file mmc1.pdf]

Supporting Information

The role of histone H3 leucine 126 in fine-tuning the copper reductase activity of nucleosomes

Nataliya P. Tod[1,#], Maria Vogelauer[1], Chen Cheng[1], Ansar Karimian[1], Stefan Schmollinger[2,##], Dimitrios Camacho[2], and Siavash K. Kurdistani[1, 3,4,*]

[1]Department of Biological Chemistry, David Geffen School of Medicine, University of California Los Angeles, Los Angeles, CA 90095, USA.

[2]Department of Molecular and Cell Biology, University of California, Berkeley, CA, 94720, USA

[3]Department of Pathology and Laboratory Medicine, David Geffen School of Medicine, University of California Los Angeles, Los Angeles, CA 90095, USA.

[4]Eli and Edythe Broad Center of Regenerative Medicine and Stem Cell Research, David Geffen School of Medicine, University of California Los Angeles, Los Angeles, CA 90095, USA.

[#]Present address: University of Texas Southwestern Medical Center, 5323 Harry Hines Blvd, Dallas, Texas 75390

[##]Present address: MSU-DOE Plant Research Laboratory & Department of Biochemistry & Molecular Biology, Michigan State University, East Lansing, Michigan, 48824

*Correspondence to: skurdistani@mednet.ucla.edu

Supporting Information:

Table S1
Figure S1
Figure S2
Figure S3
Figure S4
Figure S5
Figure S6

Table S1. Yeast strains used in this study.

Strain Name	Abbreviated Name	Genotype	Reference
**OCY1131**	WT	*MAT*a *his3Δ1 leu2Δ0 met15Δ0 ura3Δ0 (hht2Δ::ura3)Δ::HHT2*	Attar *et al.* 2020
**NTY062**	*H3^{L126M}*	*MAT*a *his3Δ1 leu2Δ0 met15Δ0 ura3Δ0 (hht2Δ::ura3)Δ::HHT2 hht2-L126M hht1-L126M*	This study
**NTY041**	*H3^{L126H}*	*MAT*a *his3Δ1 leu2Δ0 met15Δ0 ura3Δ0 (hht2Δ::ura3)Δ::HHT2 hht1-L126H hht2-L126H*	This study
**OCY2621**	*ctr1Δ*	*MAT*a *his3Δ1 leu2Δ0 met15Δ0 ura3Δ0 (hht2Δ::ura3)Δ::HHT2, ctr1Δ*	Attar *et al.* 2020
**NTY108**	*H3^{L126M}ctr1Δ*	*MAT*a *his3Δ1 leu2Δ0 met15Δ0 ura3Δ0 (hht2Δ::ura3)Δ::HHT2 hht2-L126M hht1-L126M, ctr1Δ*	This study
**NTY079**	*ccs1Δ*	*MAT*a *his3Δ1 leu2Δ0 met15Δ0 ura3Δ0 (hht2Δ::ura3)Δ::HHT2, ccs1Δ::HphMX4*	This study
**NTY081**	*H3^{L126M}ccs1Δ*	*MAT*a *his3Δ1 leu2Δ0 met15Δ0 ura3Δ0 (hht2Δ::ura3)Δ::HHT2 hht2-L126M hht1-L126M, ccs1Δ::HphMX4*	This study

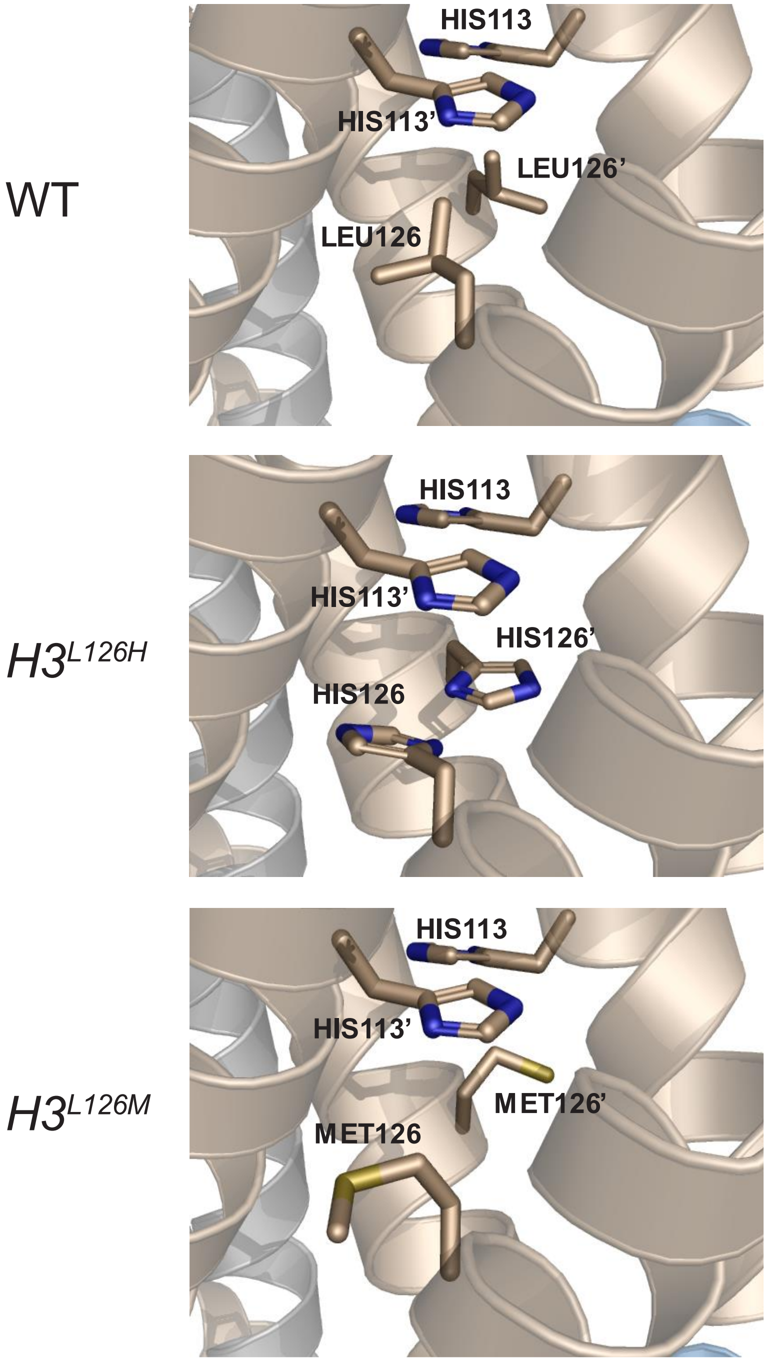

Supplement Figure S1. Mutation of H3L126 to methionine or histidine likely causes minimal steric clashes. The amino acid residues H3H113 and H3L126 at the putative active site in the H3-H3’ interface from the yeast nucleosome crystal structure (PDB file 1ID3) or the mutated amino acids, H126 or M126, are shown. Mutations were modeled using the Pymol imaging software. Using Molprobity (37) , the steric clashes for M126 ranged from 0.46 to 0.54Å and for H126 from 0.43 to 0.57Å. The range for all residues was 0.4 to 1.12Å. Rotamers with least steric hindrance were chosen for representation.

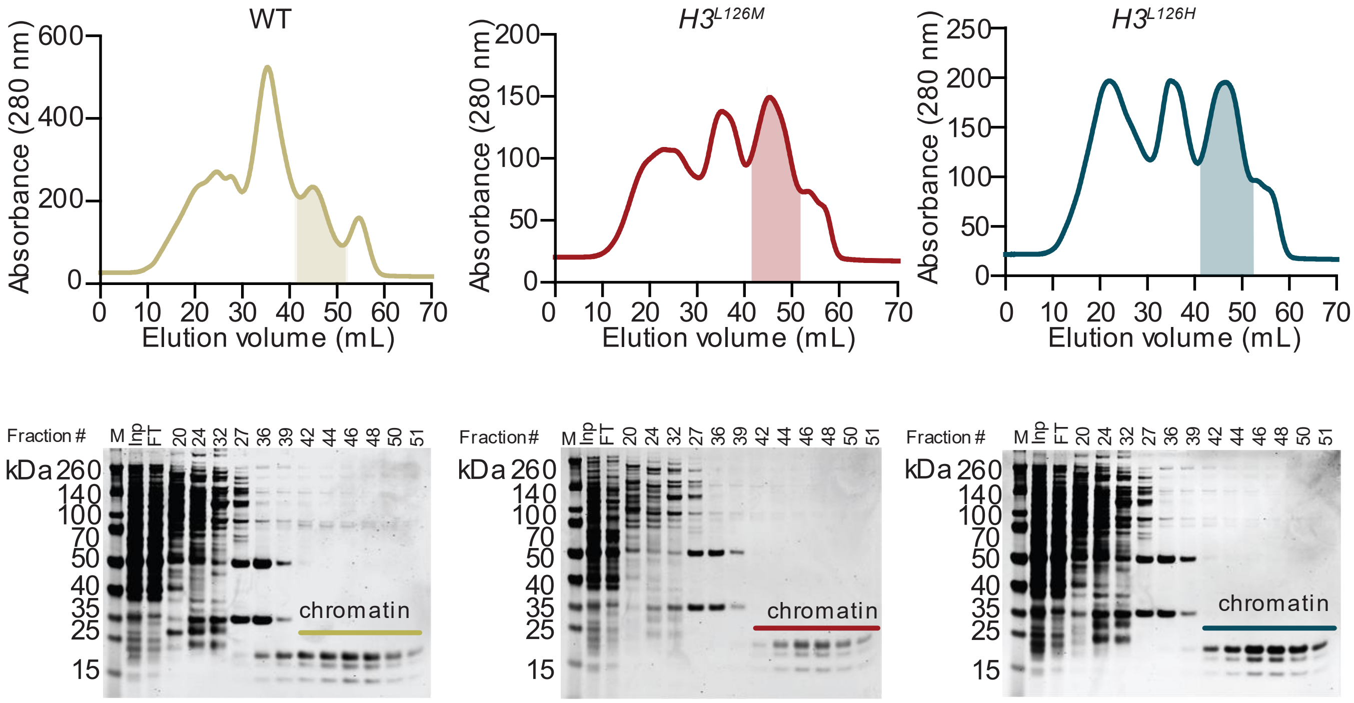

Supplement Figure S2. The overall distribution of nucleosomal fragments were comparable between WT and H3L126 mutant strains. *Upper row*: representative chromatograms display the elution profiles from the respective strains. The shaded regions highlight the fractions encompassing nucleosomes. *Lower row*: Coomassie-stained polyacrylamide gel electrophoresis analysis of the representative eluted fractions. For subsequent analysis, the fractions containing nucleosomes and indicated as "chromatin" were pooled together.

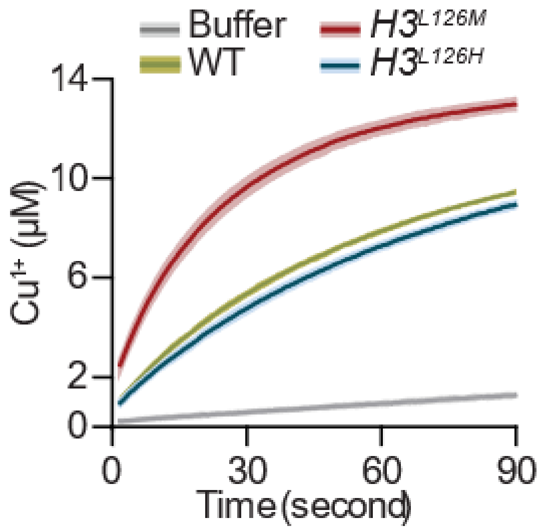

Supplement Figure S3. Mutations in L126 affect the nucleosome copper reductase activity. Progress curves of Cu^{2+} reduction by nucleosomes isolated form WT, *H3*^{L126H} or *H3*^{L126M} strains or buffer using $CuSO_4$-Tricine pH 7 at a ratio of 1:8 as substrate. Lines and shading represent the mean ± standard deviation (SD) of three assays.

A

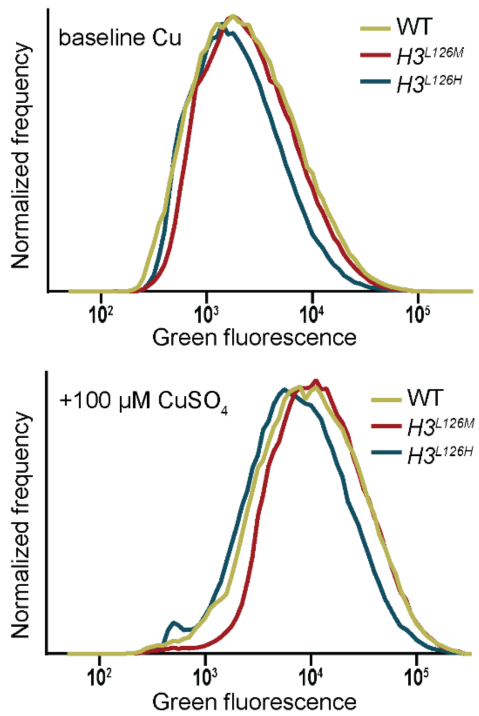

B

GO molecular function among differentially expressed genes

Upregulated in *H3*^{L126M} vs WT:
No enriched GO term

Downregulated in *H3*^{L126M} vs WT:
GO:0003993 - acid phosphatase activity p=0.00245

Upregulated in *H3*^{L126H} vs WT:
No enriched GO term

Downregulated in *H3*^{L126H} vs WT:
GO:0042578 - phosphoric ester hydrolase activity p=0.0204

Supplement Figure S4. H3L126 mutations regulate Cu^{1+}-dependent transcriptional activity of Cup2. (**A**) Average flow cytometry distributions of cells containing the p$^{(CUP1)}$-GFP plasmid grown in oxidative media lacking uracil (SCEG-ura), both in the absence (upper panels) and presence of an additional 100 µM CuSO4 (lower panel), from eight experiments. Baseline copper concentration in SCEG-ura is ~0.16 µM. Note that *CUP1* is a target gene of Cup2, which functions by binding Cu^{1+} and activating the expression of its target genes. (**B**) Gene ontology (GO) enrichment analysis in molecular function category of the indicated group of genes. The data are from cells grown in fermentative media.

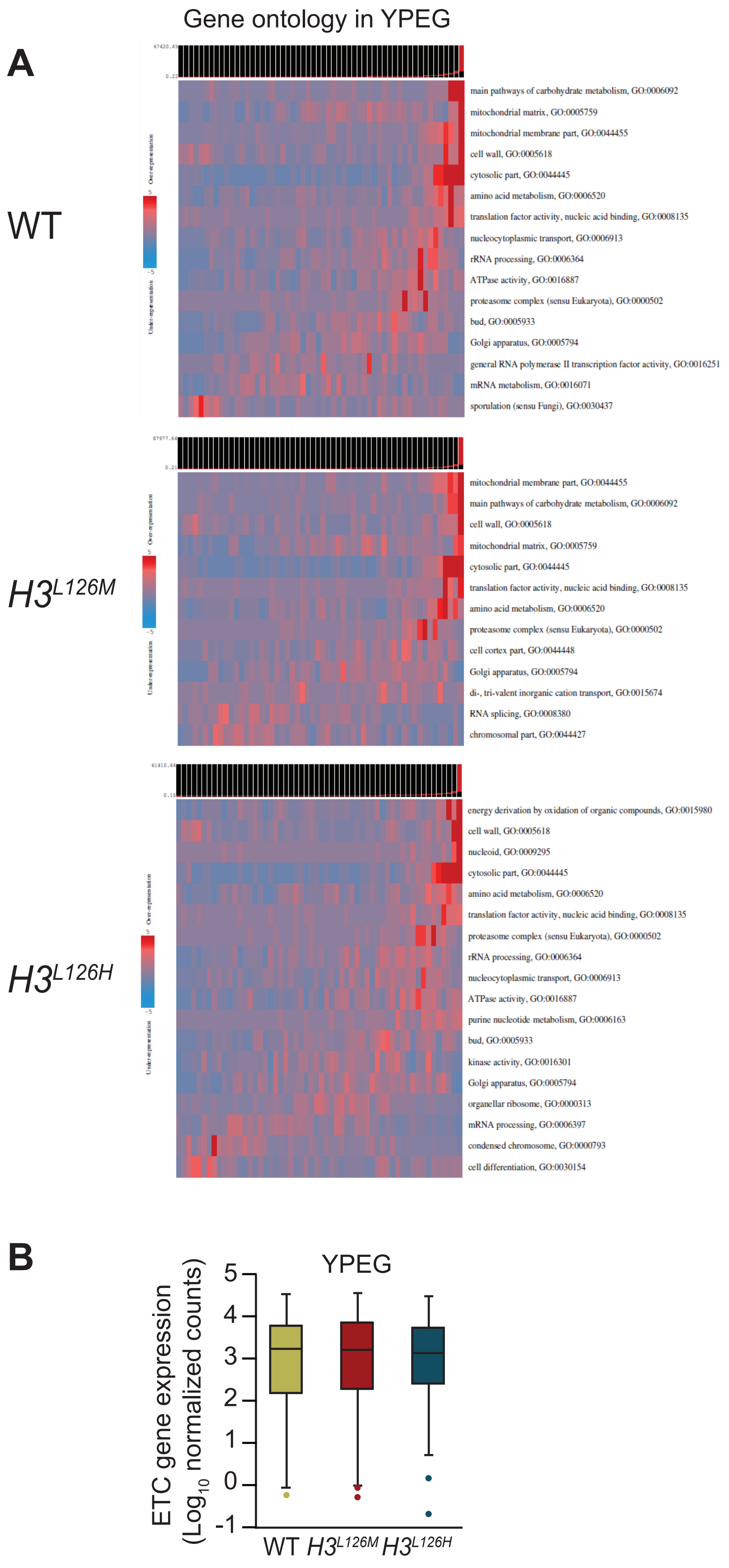

Gene ontology in YPEG
A
WT
main pathways of carbohydrate metabolism, GO:0006092
mitochondrial matrix, GO:0005759
mitochondrial membrane part, GO:0044455
cell wall, GO:0005618
cytosolic part, GO:0044445
amino acid metabolism, GO:0006520
translation factor activity, nucleic acid binding, GO:0008135
nucleocytoplasmic transport, GO:0006913
rRNA processing, GO:0006364
ATPase activity, GO:0016887
proteasome complex (sensu Eukaryota), GO:0000502
bud, GO:0005933
Golgi apparatus, GO:0005794
general RNA polymerase II transcription factor activity, GO:0016251
mRNA metabolism, GO:0016071
sporulation (sensu Fungi), GO:0030437
H3L126M
mitochondrial membrane part, GO:0044455
main pathways of carbohydrate metabolism, GO:0006092
cell wall, GO:0005618
mitochondrial matrix, GO:0005759
cytosolic part, GO:0044445
translation factor activity, nucleic acid binding, GO:0008135
amino acid metabolism, GO:0006520
proteasome complex (sensu Eukaryota), GO:0000502
cell cortex part, GO:0044448
Golgi apparatus, GO:0005794
di-, tri-valent inorganic cation transport, GO:0015674
RNA splicing, GO:0008380
chromosomal part, GO:0044427
H3L126H
energy derivation by oxidation of organic compounds, GO:0015980
cell wall, GO:0005618
nucleoid, GO:0009295
cytosolic part, GO:0044445
amino acid metabolism, GO:0006520
translation factor activity, nucleic acid binding, GO:0008135
proteasome complex (sensu Eukaryota), GO:0000502
rRNA processing, GO:0006364
nucleocytoplasmic transport, GO:0006913
ATPase activity, GO:0016887
purine nucleotide metabolism, GO:0006163
bud, GO:0005933
kinase activity, GO:0016301
Golgi apparatus, GO:0005794
organellar ribosome, GO:0000313
mRNA processing, GO:0006397
condensed chromosome, GO:0000793
cell differentiation, GO:0030154
B
YPEG
ETC gene expression
(Log10 normalized counts)
5
4
3
2
1
0
-1
WT
H3L126M
H3L126H

Supplement Figure S5. H3L126 mutations do not result in major gene expression patterns in oxidative media compared to WT. (**A**) Heatmaps show enriched gene ontology groups in expression profiles of WT, $H3^{L126M}$ or $H3^{L126H}$ strains grown in oxidative media. The significance of enrichment is color coded according to the scale shown to the left (red – relative enrichment, blue – relative depletion) (**B**) Average mRNA expression of genes involved in mitochondrial electron transport chain (ETC) are depicted for the indicated strains from cells grown in oxidative media.

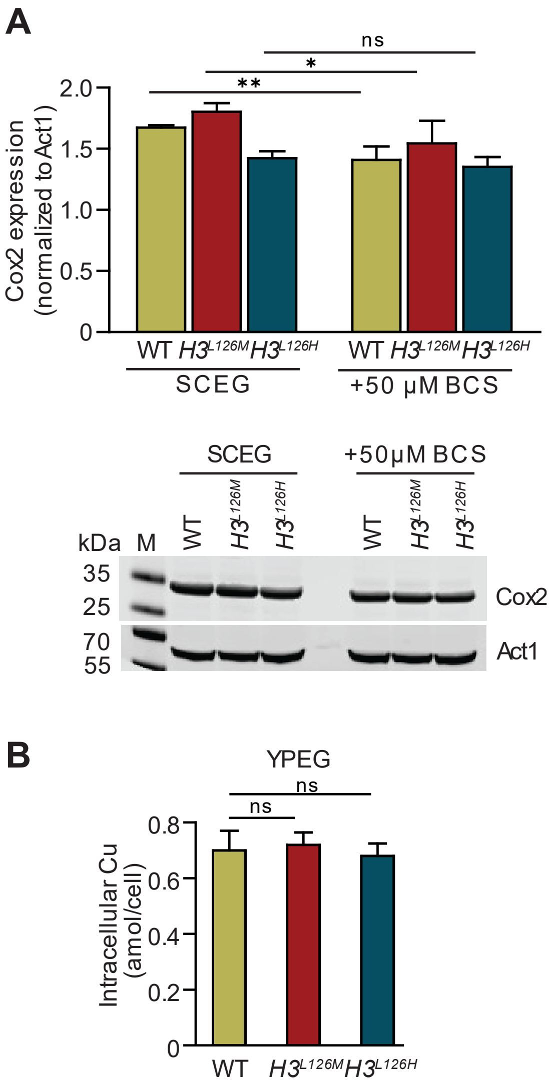

Supplement Figure S6. Functional impact of H3L126 mutation on copper-dependent activities is not due to gene expression differences or total intracellular levels of copper. (**A**) Cox2 protein expression in WT, *H3*^{L126M} and *H3*^{L126H} strains in oxidative media (SCEG) or oxidative media with low copper levels (SCEG + 50 µM BCS). Upper bar graph reports the average Cox2 expression normalized to Act1 expression as analyzed by Western blotting. Bar graphs are means ± SD from three experiments. Lower panel displays a representative Western blot. Statistically significant differences as determined by one-tailed t-test are indicated by asterisks: *p<0.05, **p<0.01, ***p<0.001. ns, not significant. (**B**) Intracellular copper content of exponentially growing strains in oxidative media as determined by ICP-MS. Bars are means ± SD from five experiments.
